# Body fatness during childhood and adolescence and breast density in young women: a prospective analysis

**DOI:** 10.1186/s13058-015-0601-4

**Published:** 2015-07-16

**Authors:** Kimberly A. Bertrand, Heather J. Baer, E. John Orav, Catherine Klifa, John A. Shepherd, Linda Van Horn, Linda Snetselaar, Victor J. Stevens, Nola M. Hylton, Joanne F. Dorgan

**Affiliations:** Channing Division of Network Medicine, Brigham and Women’s Hospital and Harvard Medical School, 181 Longwood Avenue, Boston, MA 02115 USA; Department of Epidemiology, Harvard T.H. Chan School of Public Health, 677 Huntington Avenue, Boston, MA 02115 USA; Division of General Internal Medicine and Primary Care, Brigham and Women’s Hospital and Harvard Medical School, 75 Francis Street, Boston, MA 02120 USA; Department of Biostatistics, Harvard T.H. Chan School of Public Health, 677 Huntington Avenue, Boston, MA 02115 USA; Dangeard Group, 740 chemin de la Commanderie St Jean de Malte, 13080 Luynes, France; Department of Radiology, University of California, 505 Parnassus Avenue, San Francisco, CA 94143 USA; Department of Preventive Medicine, Northwestern University, 680 North Lake Shore Drive, Chicago, IL 60611 USA; Department of Epidemiology, University of Iowa College of Public Health, 145 North Riverside Drive, Iowa City, IA 52242 USA; Kaiser Permanente Center for Health Research, 3800 North Interstate Avenue, Portland, OR 97227 USA; Department of Epidemiology and Public Health, University of Maryland School of Medicine, 655 West Baltimore Street, Baltimore, MD 21201 USA

## Abstract

**Introduction:**

Overweight and obesity in childhood and adolescence are associated with reduced breast cancer risk, independent of adult body mass index (BMI). These associations may be mediated through breast density.

**Methods:**

We prospectively examined associations of early life body fatness with adult breast density measured by MRI in 182 women in the Dietary Intervention Study in Children (DISC) who were ages 25–29 at follow-up. Height, weight, and other factors were measured at baseline (ages 8–10) and annual clinic visits through adolescence. We used linear mixed-effects models to quantify associations of percent breast density and dense and non-dense breast volume at ages 25–29 with quartiles of age-specific youth body mass index (BMI) Z-scores, adjusting for clinic, treatment group, current adult BMI, and other well-established risk factors for breast cancer and predictors of breast density.

**Results:**

We observed inverse associations between age-specific BMI Z-scores at all youth clinic visits and percent breast density, adjusting for current adult BMI and other covariates (all *p* values <0.01). Women whose baseline BMI Z-scores (at ages 8–10 years) were in the top quartile had significantly lower adult breast density, after adjusting for current adult BMI and other covariates [least squares mean (LSM): 23.4 %; 95 % confidence interval (CI): 18.0 %, 28.8 %] compared to those in the bottom quartile (LSM: 31.8 %; 95 % CI: 25.2 %, 38.4 %) (*p* trend <0.01). Significant inverse associations were also observed for absolute dense breast volume (all *p* values <0.01), whereas there were no clear associations with non-dense breast volume.

**Conclusions:**

These results support the hypothesis that body fatness during childhood and adolescence may play an important role in premenopausal breast density, independent of current BMI, and further suggest direct or indirect influences on absolute dense breast volume.

**Clinical Trials Registration Number:**

NCT00458588; April 9, 2007

**Electronic supplementary material:**

The online version of this article (doi:10.1186/s13058-015-0601-4) contains supplementary material, which is available to authorized users.

## Introduction

Greater body fatness at young ages is associated with substantially decreased risk of breast cancer both in premenopausal and postmenopausal women [1–3], and this association is independent of adult body mass index (BMI) and other established breast cancer risk factors [3]. Body fatness at young ages may affect breast cancer risk through breast density, an established biomarker for breast cancer risk [4] that refers to the proportion of fibroglandular tissue (versus adipose tissue) in the breast [5].

Although adult BMI is inversely related to breast density, there has been little research focusing on the role of body fatness at younger ages [6–11]. Since most breast development occurs during puberty, body fatness during this time period could have an important impact on breast morphology and consequently breast density later in life, influencing breast cancer risk through this pathway. Results from recent studies [6, 7, 9, 10, 12] support the hypothesis that body fatness at young ages may influence breast density later in life. Because mammography is not generally recommended in younger women given risks associated with radiation exposure, few studies have evaluated these associations in young premenopausal women. Moreover, recent evidence suggests that absolute dense and non-dense areas may have independent effects on cancer risk [13–15]. Understanding the possible differential associations of early life body fatness with absolute density phenotypes could provide additional insight into possible mechanisms underlying these associations.

To examine whether body fatness during childhood and adolescence is associated with percent breast density and absolute dense and non-dense breast volume in young adult women, we conducted a prospective analysis among women in the Dietary Intervention Study in Children 2006 (DISC06) Follow-up Study.

## Methods

### Study population

The original DISC was a multicenter, randomized controlled trial to examine the safety and efficacy of a dietary intervention to reduce serum low-density lipoprotein cholesterol (LDL-C) in children [16–19]. Briefly, between 1988 and 1990, 663 healthy, prepubertal 8–10-year-old children, including 301 girls, with elevated LDL-C were recruited to six clinical centers and randomized to a behavioral dietary intervention or usual care control group. Children participated in annual clinic visits until the trial was terminated in 1997, when the average age of participants was 16.7 years, due to a lack of treatment effect on LDL-C. In 2006–2008 when participants were 25–29 years old, the DISC06 Follow-up Study was conducted to evaluate longer-term effects of the dietary intervention on biomarkers associated with breast cancer risk in female participants. At the time of the original DISC, assent was obtained from participants; their parents/guardians provided informed consent before randomization. Participants were re-consented before the DISC06 follow-up visit. The original DISC protocol was approved by an NHLBI-appointed independent data and safety monitoring committee and institutional review boards (IRBs) at all participating clinical centers and the data coordinating center. The DISC06 Follow-up Study protocol was approved by IRBs at the Fox Chase Cancer Center, participating clinical centers, and the data coordinating center (please see Acknowledgments).

For this analysis, participants included 182 women who enrolled in the original DISC between 1988 and 1990, when they were ages 8–10 years, and also participated in the DISC 2006 (DISC06) Follow-up Study, when they were ages 25–29 years. Women who were pregnant or breastfeeding within 12 weeks (n = 30) before their clinic visit, had breast implants or reduction surgery (n = 16), or whose magnetic resonance imaging (MRI) scan was missing or of poor quality (n = 32) were excluded.

### Data collection

During the original DISC, height and weight were measured at baseline and annual clinic visits by trained study staff blinded to treatment assignment. Specifically, height was measured using a stadiometer and weight was measured on an electronic or beam balance scale. Each measurement was made twice. A third measurement was taken if the first two measurements were not within allowable tolerances (0.5 cm for height and 0.2 kg for weight) and the two closest values were averaged. BMI was calculated as weight (kg)/height (m^2^) and expressed as a Z-score relative to Centers for Disease Control and Prevention (CDC) 2000 growth charts [20]. Information on demographic and other characteristics, including medical history and reproductive factors, was ascertained on annual questionnaires. More extensive data, including three 24-h dietary recalls and physical activity assessments, were collected at baseline and years 1, 3, 5 and last childhood visits [21, 22].

DISC06 follow-up visits took place at the original six DISC clinics and were scheduled during the luteal phase of the menstrual cycle when possible (>85 % of visits occurred within 14 days of onset of next menses). Information was updated and percent breast density was assessed following a standardized protocol by non-contrast MRI. Equipment standards at each site were consistent with American College of Radiology guidelines for breast MRI [23] and required that imaging be performed using a whole-body MRI scanner of 1.5 Tesla or higher field strength and a dedicated breast imaging radiofrequency coil. A standard image-acquisition protocol was prescribed consisting of two pulse sequences performed in both the transaxial and coronal orientations with a 32–40-cm field of view for bilateral coverage: a three-dimensional fast gradient echo sequence without fat suppression, and a three-dimensional fast gradient echo sequence with fat suppression.

To ensure accuracy and uniformity of data acquisition at the different clinical centers, MRI technologists at the sites were individually trained (by CK) to recognize and correct failures due to incomplete fat suppression, motion artifacts, and inadequate breast coverage. In addition, acceptable image quality on three volunteers was required for site certification. Participant scans that were inaccurate due to artifacts, motion or technique were excluded (n = 21).

All MRI image data were processed at the University of California, San Francisco by CK using customized software to identify the chest wall-breast tissue boundary and skin surface, and to separate breast fibroglandular and fatty tissue using a segmentation method based on fuzzy C-means clustering [24]. Total volumes of fibroglandular and fatty tissue were computed separately for each breast and averaged for analysis. Outcomes of interest were percent dense breast tissue (ratio of fibroglandular volume to total volume of the breast), total volume of dense tissue (fibroglandular volume), and total volume of non-dense tissue (fatty breast tissue volume).

### Statistical analyses

We used linear mixed-effects models to examine the association between percent breast density at ages 25–29 (outcome) and body mass index (BMI) Z-scores at baseline (ages 8–10 years) (predictor). The analysis was repeated, changing the predictor to BMI Z-score at each annual clinic visit during childhood and adolescence.

For our predictor, body fatness during childhood, we created quartiles of age- and female-specific BMI Z-scores computed from CDC growth charts [20]. A linear mixed-effects model was fit by maximum likelihood with clinic included as a random effect and empirical (“robust”) standard errors to allow for correlated outcomes within clinics. We considered potential confounding by the following variables: current BMI [25], age at menarche, number of live births [26], duration of hormone use [26], menstrual cycle day, height [25], alcohol consumption, race, education, moderate to intense physical activity [22], smoking status, and family history of breast cancer. Fully adjusted multivariable models include fixed-effect covariates that are well-established risk factors for breast cancer (i.e., age at menarche, duration of hormone use, race, and alcohol consumption) and those that were statistically significantly associated with density when included (current BMI, current BMI-squared, number of live births, education, smoking status, and family history of breast cancer). Categorical covariates were categorized as shown in Table 1.

**Table 1:** Participant characteristics (at the DISC06 follow-up visit)

Descriptive characteristic	n	Mean (SD) or %
Age (y)	182	27.2 (1.0)
Percent dense breast volume (%)	182	27.6 (20.5)
Absolute dense breast volume (cm^3^)	182	104.2 (70.6)
Absolute non-dense breast volume (cm^3^)	182	413.5 (364.3)
Body mass index (BMI) (kg/m^2^)	182	24.4 (5.4)
Height (cm)	182	165.3 (6.4)
BMI Z-score at 8–10 years old	182	0.23 (0.90)
Age at menarche (y)	182	12.9 (1.3)
Duration of hormonal contraceptive use (y)^a^	171	5.6 (3.5)
Race		
White	164	90.1 %
Non-white	18	9.9 %
Education		
High school, vocational, or technical school	18	9.9 %
Some college	44	24.2 %
College/Bachelor’s	95	52.2 %
Graduate school	25	13.7 %
Number of live births		
0	129	73.1 %
1	30	16.5 %
2+	23	12.6 %
Ever breast fed (among parous)		
Yes	40	75.4 %
No	13	24.5 %
Hormonal contraceptive use		
Never	11	6.0 %
Former	66	36.3 %
Current	105	57.7 %
Family history of breast cancer		
Yes	7	3.9 %
No	171	96.1 %
Alcohol consumption		
Never/former	16	8.8 %
Current, <3 drinks per week	71	39.0 %
Current, 3–<6 drinks per week	33	18.1 %
Current, 6– <10 drinks per week	40	22.0 %
Current, 10+ drinks per week	22	12.1 %
Smoking history		
Never	100	55.0 %
Former	38	20.9 %
Current	44	24.2 %
Treatment assignment		
Intervention	87	47.8 %
Usual care	95	52.2 %

^a^Among current or former hormonal contraceptive users

The association between quartiles of childhood BMI Z-scores and adult breast density was quantified by adjusted least squares means and 95 % confidence intervals (CIs). Tests for trend and estimates of differences in adult breast density per youth BMI Z-score (i.e., per standard deviation of BMI) were based on models including childhood or adolescent BMI Z-score as a continuous variable.

The same analytic approach was used to examine the alternative outcomes of absolute dense and non-dense tissue; to improve normality, we applied a natural log-transformation to absolute measures of density phenotypes and for consistency with prior literature. Because of the log-transformation, we applied Duan’s “smearing estimate” to back-transform estimates to original (untransformed) units of cm^3^; this method adjusts for bias arising in retransformation of estimates from nonparametric or generalized linear models [27].

In secondary, post hoc analyses, we explored the possible independent associations of childhood and adolescent BMI at different ages with adult breast density. First, we fit a model that mutually adjusted for BMI Z-score at each age. Measures of BMI during childhood and adolescence were strongly correlated, which could lead to multicollinearity or model instability. To evaluate possible multicollinearity, we fit ordinary least squares regression models, including all BMI Z-scores during youth visits and BMI at follow-up to calculate variance inflation factors (VIFs). All VIFs for the age-specific BMI variables were <10, suggesting that multicollinearity is not a substantial concern in these analyses [28–32]. Second, we cross-classified categories of lean versus heavy body type in childhood/adolescence versus follow-up (dichotomized at the median BMI Z-scores: 0.38 and −0.25, respectively) and compared average percent breast density among women who were lean at both time points with those who were heavy at both time points or whose body fatness changed (e.g., lean to heavy or heavy to lean).

## Results

Among the 182 women, the mean age was 27.2 years and mean adult BMI was 24.4 kg/m^2^ at the DISC06 follow-up visit. On average, mean BMI Z-score at baseline at ages 8–10 years was 0.23. The majority of women were white (90.1 %), had high levels of education (>90 % had at least some college), and were nulliparous (73.1 %) at the time of follow-up. Mean percent dense breast volume was 27.6 % (standard deviation, 20.5) (Table 1; full distributions of breast density metrics and BMI are shown in Figures S1a and Figure S1b in Additional file 1). Other characteristics of the study population are shown in Table 1. Table S1 in Additional file 2 shows selected descriptive characteristics of the study population over each of the five youth DISC clinic visits with more extensive data collection; Table S2 in Additional file 2 shows the high correlations between BMI measured at the various study time points; Table S3 in Additional file 2 shows correlations between BMI at each visit with breast density measures.

**Fig. 1:**
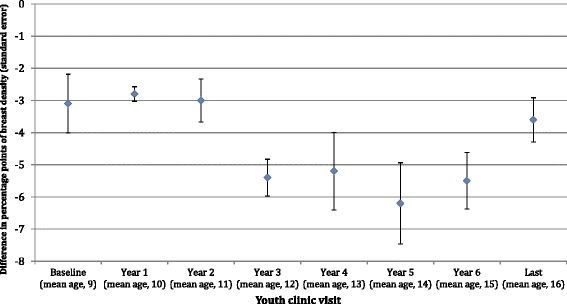
Difference in percent dense volume at the DISC06 follow-up visit per unit increase in body mass index (BMI) Z-score, by youth clinic visits

We observed strong, significant inverse associations between the age-specific BMI Z-score at all clinic visits during childhood and adolescence and percent breast density in adulthood (all *p* values <0.01) (Fig. 1). Because of similar patterns in associations across youth clinic visits, in Table 2 we present results from simple and multivariable adjusted models only for baseline, Year 3, Year 5, and last clinic visit. Associations were attenuated upon adjustment for current adult BMI (Model 2), but remained statistically significant. For example, women whose BMI Z-scores, measured when they were between 8 and 10 years old, were in the top quartile of the cohort had significantly lower BMI-adjusted breast density (least squares mean: 23.7 %; 95 % confidence interval: 19.3 %, 28.0 %) compared to those in the bottom quartile (least squares mean: 31.3 %; 95 % confidence interval: 25.0 %, 37.6 %) (*p* trend <0.01). While number of live births, duration of hormonal contraceptive use, educational attainment, smoking status, and family history of breast cancer were significant predictors of percent breast density in the DISC06 cohort, inclusion of these variables in the models did not substantially change estimates of the associations between childhood and adolescent BMI and adult breast density. Further, we did not observe evidence of confounding by other suspected predictors of breast density, such as age at menarche, race, or alcohol consumption (Table 2; Model 3). In these models, the decrease in percent breast density ranged from 3.1 to 6.2 percentage points per 1-unit increase in childhood or adolescent BMI Z-score (i.e., per standard deviation of childhood or adolescent BMI).

We observed the strongest associations for the Year 5 clinic visit when girls were ages 13–15, during puberty for most of them. Therefore, in secondary analyses, we fit a multivariable model including BMI Z-score at the five youth clinic visits and current adult BMI Z-score to assess the possible independent associations of BMI at various ages in childhood and adolescence with young adult percent breast density. In these models, the strongest inverse associations were observed for BMI Z-score at the Year 3 visit (ages 11–13) and current BMI Z-score. These variables were statistically significantly independently associated with breast density (differences of −6.7 and −17.0 percentage points per standard deviation of BMI, respectively; *p* values <0.01 in mutually adjusted model).

**Fig. 2:**
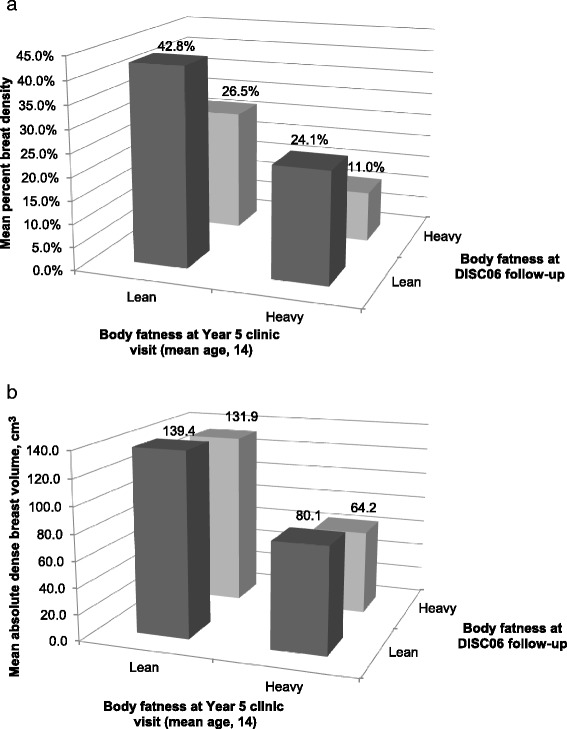
**a** Mean percent breast density by body fatness in adolescence and at follow-up. **b** Mean absolute dense breast volume by body fatness in adolescence and at follow-up

To further explore changes in BMI over time associated with breast density, we considered cross-classified categories of lean versus heavy in childhood/adolescence (Year 5 visit) versus adult follow-up and observed that women who were heavier at both time periods had the lowest breast density (mean =11.0 %) while those who were leaner at both time periods had the highest breast density (mean = 42.8 %). Those who were lean in childhood and heavy in adulthood and vice versa had intermediate values for breast density (26.5 % and 24.1 %, respectively) (Fig. 2a).

In separate analyses, we observed significant inverse associations between childhood/adolescent BMI and adult absolute dense breast volume (*p* values <0.01) (Table 3). In these analyses, there was little evidence of confounding by adult BMI or any of the other covariates. Each unit increase in youth BMI Z-score was associated with a 24–38 % lower dense breast volume. The strongest association was for BMI in Year 5 at ages 13–15. Indeed, in mutually adjusted models, BMI at this time point had the lowest *p* value; however, none were statistically significant when all were included in the same model (data not shown). Considering joint categorizations of body fatness in childhood/adolescence (Year 5) and adult follow-up, we observed that women who were heavy in adolescence had lower mean absolute dense tissue volume while women who were lean in adolescence had higher absolute dense breast volume, regardless of their body fatness in adulthood (Fig. 2b). There were no clear associations between childhood or adolescent BMI and absolute non-dense (fat) breast volume, after adjusting for current adult BMI (Table 4).

**Table 2:** Least squares mean and 95 % confidence interval (CI) for percent dense breast volume at the DISC06 follow-up visit according to quartile of age-specific youth body mass index (BMI) Z-score

Quartile of age-specific youth BMI Z-score:		Q1	Q2	Q3	Q4	Change in adult percent breast density per unit youth BMI Z-score	*p* value^*^
BMI at baseline visit (ages 8–10)	Model 1	40.9 (34.6, 47.2)	32.9(30.0, 35.8)	23.6 (19.6, 27.6)	13.1 (9.4, 16.8)	−11.1	<0.01
n = 182	Model 2	31.3 (25.0, 37.6)	28.9 (26.6, 31.3)	26.7 (22.1, 31.3)	23.7 (19.3, 28.0)	−2.7	<0.01
	Model 3	31.8 (25.2, 38.4)	29.0 (26.0, 32.0)	26.2 (22.2, 30.1)	23.4 (18.0, 28.8)	−3.1	<0.01
BMI at Year 3 visit (ages 11–13)	Model 1	43.3 (36.7, 50.0)	34.6 (27.5, 41.8)	19.7 (16.8, 22.6)	12.8 (9.9, 15.7)	−12.9	<0.01
n = 170	Model 2	33.8 (27.6, 40.0)	30.8 (24.8, 36.8)	24.1 (21.5, 26.8)	21.8 (18.6, 24.9)	−5.1	<0.01
	Model 3	34.5 (28.2, 40.7)	30.4 (26.5, 34.2)	25.0 (22.3, 26.9)	21.1 (18.6, 23.5)	−5.4	<0.01
BMI at Year 5 visit (ages 13–15)	Model 1	44.5 (38.7, 50.2)	39.0 (33.0, 44.9)	18.7 (15.0, 22.4)	9.9 (6.8, 13.0)	−15.5	<0.01
n = 153	Model 2	35.4 (30.1, 40.8)	31.8 (26.4, 37.2)	22.7 (18.6, 26.9)	21.6 (14.9, 28.2)	−6.2	<0.01
	Model 3	34.5 (29.3, 39.7)	31.6 (19.3, 28.5)	23.9 (19.3, 28.5)	21.3 (14.8, 27.7)	−6.2	<0.01
BMI at last visit (ages 15–17)	Model 1	44.7 (37.4, 51.9)	36.0 (30.9, 41.1)	23.9 (19.9, 28.0)	11.0 (10.0, 12.0)	−14.0	<0.01
n = 159	Model 2	32.3 (26.6, 38.0)	30.4 (23.4, 37.5)	29.0 (24.6, 33.4)	23.8 (22.0, 25.5)	−3.4	<0.01
	Model 3	32.1 (26.0, 38.1)	30.7 (24.6, 36.8)	29.0 (23.2, 34.9)	23.3 (20.8, 25.8)	−3.6	<0.01

Cut points for quartiles are: baseline Z-BMI −1.9 to 0.47, −0.46 to 0.27, 0.28 to 0.98, 0.98 to 1.85; Year 3 Z-BMI −2.4 to 0.37, −0.34 to 0.34, 0.35 to 1.14, 1.15 to 2.17; Year 5 visit −2.2 to 0.21, −0.21 to 0.37, 0.38 to 1.09, 1.53 to 2.19; Last visit −2.25 to 0.26, −0.23 to 0.30, 0.33 to 0.98, 0.99 to 2.13

Model 1 Least squares means estimated from linear mixed-effects models including clinic as a random effect and treatment group as a fixed effect

Model 2 Least squares means estimated from linear mixed-effects models including clinic as a random effect and adjusted for treatment group and current adult BMI (continuous, kg/m^2^) as fixed effects

Model 3 Least squares means estimated from linear mixed-effects models including clinic as a random effect and adjusted for treatment group, current adult BMI (continuous, kg/m^2^), number of live births, duration of hormone use, age at menarche, race, education, alcohol consumption, smoking status, and family history of breast cancer, as fixed effects (4 missing)

^*^Test for trend

**Table 3:** Mean and 95 % confidence interval (CI) for absolute dense breast volume (cm^3^) at the DISC06 follow-up visit according to quartile of age-specific youth body mass index (BMI) Z-score

Quartile of age-specific youth BMI Z-score:		Q1	Q2	Q3	Q4	% change in dense breast volume per unit youth BMI Z-score	*p* value^*^
BMI at baseline visit (ages 8–10)	Model 1	155.5 (132.8, 182.1)	110.6 (98.2, 124.5)	102.2 (80.6, 129.7)	63.1 (44.9, 88.9)	−28.1 %	<0.01
n = 182	Model 2	142.8 (117.5, 173.5)	107.7 (95.5, 121.4)	105.9 (83.2, 134.8)	68.2 (47.1, 98.9)	−22.9 %	<0.01
	Model 3	145.0 (116.1, 181.2)	109.4 (95.0, 126.1)	95.6 (74.5, 122.7)	64.9 (45.7, 92.0)	−25.2 %	<0.01
BMI at Year 3 visit (ages 11–13)	Model 1	154.1 (140,9, 168.4)	127.9 (103.6, 157.7)	91.0 (77.8, 106.3)	59.8 (38.0, 94.0)	−31.6 %	<0.01
n = 170	Model 2	142.2 (124.4, 162.6)	126.0 (100.3, 158.3)	95.0 (78.6, 114.9)	63.1 (38.0, 104.7)	−29.5 %	<0.01
	Model 3	147.2 (118.2, 183.2)	120.4 (103.7, 139.7)	89.4 (74.4, 107.4)	58.5 (38.1, 89.8)	−32.3 %	<0.01
BMI at Year 5 visit (ages 13–15)	Model 1	161.2 (131.4, 197.7)	128.7 (118.1, 140.4)	82.9 (69.1, 99.5)	56.1 (34.6, 90.8)	−35.6 %	<0.01
n = 153	Model 2	166.4 (132.4, 209.2)	133.5 (120.1, 148.3)	84.6 (69.9, 102.3)	51.4 (26.5, 99.7)	−36.9 %	<0.01
	Model 3	156.2 (129.5, 188.5)	121.7 (105.0, 141.0)	85.2 (67.9, 107.0)	48.8 (28.0, 85.0)	−38.1 %	<0.01
BMI at last visit (ages 15–17)	Model 1	152.8 (126.9, 183.8)	123.7 (97.2, 157.4)	97.6 (70.0, 135.4)	65.9 (52.8, 82.2)	−28.8 %	<0.01
n = 159	Model 2	138.9 (110.0, 175.5)	120.1 (89.6, 160.9)	102.5 (75.2, 139.8)	70.6 (58.7, 84.9)	−23.7 %	<0.01
	Model 3	130.3 (97.6, 173.8)	118.8 (88.4, 159.6)	97.6 (73.6, 129.3)	66.2 (52.1, 84.0)	−23.7 %	<0.01

Model 1 Means estimated from linear mixed-effects models including clinic as a random effect and treatment group as a fixed effect

Model 2 Means estimated from linear mixed-effects models including clinic as a random effect and adjusted for treatment group and current adult BMI as fixed effects

Model 3 Means estimated from linear mixed-effects models including clinic as a random effect and adjusted treatment group, current adult BMI, number of live births, duration of hormone use, age at menarche, race, education, alcohol consumption, smoking status, family history of breast cancer, and log-non-dense area as fixed effects

^*^Test for trend

**Table 4:** Mean and 95 % confidence interval (CI) for absolute non-dense breast volume (cm^3^) at the DISC06 follow-up visit according to quartile of age-specific youth body mass index (BMI) Z-score

Quartile of age-specific youth BMI Z-score:		Q1	Q2	Q3	Q4	% change in non-dense breast volume per unit youth BMI Z-score	*p* value^*^
BMI at baseline visit (ages 8–10)	Model 1	252.8 (209.2, 305.5)	308.4 (242.4, 392.5)	462.7 (425.1, 503.7)	656.4 (573.4, 751.6)	47.7 %	<0.01
n = 182	Model 2	356.2 (294.2, 431.1)	328.2 (300.0, 359.0)	329.5 (282.9, 383.7)	285.2 (244.6, 332.5)	−8.6 %	0.04
	Model 3	351.4 (291.1, 424.2)	325.9 (290.9, 351.3)	290.2 (243.5, 345.8)	290.2 (243.5, 345.8)	−8.6 %	<0.01
BMI at Year 3 visit (ages 11–13)	Model 1	217.8 (166.7, 284.6)	292.8 (226.3, 378.8)	531.7 (430.4, 656.9)	624.5 (581.7, 670.5)	60.0 %	<0.01
n = 170	Model 2	317.4 (244.5, 412.1)	322.6 (272.5, 381.9)	352.9 (321.8, 387.1)	299.5 (262.3, 341.9)	−3.0 %	0.64
	Model 3	313.4 (241.2, 407.3)	310.2 (267.3, 360.1)	342.9 (313.0, 275.7)	311.0 (278.5, 347.3)	−1.0 %	0.80
BMI at Year 5 visit (ages 13–15)	Model 1	203.4 (173.5, 238.5)	214.7 (166.8, 276.5)	502.3 (424.2, 594.8)	772.1 (652.2, 913.9)	84.0 %	<0.01
n = 153	Model 2	318.0 (263.0, 384.5)	305.3 (247.2, 377.1)	365.7 (321.7, 415.8)	287.6 (242.6, 340.9)	−3.0 %	0.71
	Model 3	310.2 (271.6, 354.3)	291.6 (247.0, 344.1)	356.3 (300.8, 421.9)	303.3 (267.9, 343.6)	0.8 %	0.85
BMI at last visit (ages 15–17)	Model 1	191.8 (154.8, 237.7)	243.4 (216.1, 274.2)	460.8 (386.1, 550.0)	753.4 (702.2, 808,3)	80.4 %	<0.01
n = 159	Model 2	328.4 (277.1, 389.3)	293.1 (261.7, 328.3)	329.1 (275.3, 393.5)	297.2 (270.4, 326.8)	−2.0 %	0.60
	Model 3	326.5 (286.6, 372.1)	286.7 (257.0, 319.9)	317.0 (258.9, 388.2)	304.4 (279.2, 331.8)	0.1 %	0.98

Model 1 Means estimated from linear mixed-effects models including clinic as a random effect and treatment group as a fixed effect

Model 2 Means estimated from linear mixed-effects models including clinic as a random effect and adjusted for treatment group and current adult BMI as fixed effects

Model 3 Means estimated from linear mixed-effects models including clinic as a random effect and adjusted treatment group, current adult BMI, number of live births, duration of hormone use, age at menarche, race, education, alcohol consumption, smoking status, family history of breast cancer, and log-dense area as fixed effects

^*^Test for trend

## Discussion

In summary, we confirmed significant inverse associations between body fatness during childhood and adolescence and percent breast density in adult women ages 25–29, independent of current adult BMI [25], consistent with most [6–11] but not all [33, 34] previous studies in this area. Significant inverse associations were observed for absolute dense breast volume (all *p* values <0.01), whereas there were no clear associations of early life body fatness with non-dense breast volume.

Early life may be a time of increased susceptibility to exposures and host factors that influence breast cancer risk [35]. Mammary gland cells undergo rapid proliferation during puberty, which could explain why exposures during this time period might be important for breast density and breast cancer risk [35–37]. The suggestion of somewhat stronger associations between childhood BMI during puberty (ages 13–15) and adult percent breast density supports the hypothesis that body fatness could influence breast cancer risk through effects on breast density. A register-based cohort study in Denmark reported strong inverse associations between youth BMI, especially at age 13, and mammographic density at a mean age of 54.6 years [38]. Further, the authors reported significant associations between BMI at age 13 and risk of breast cancer that were attenuated upon additional adjustment for mammographic density, suggesting that density could mediate associations between early life BMI and breast cancer risk [38]. In contrast, however, the possible mediating effect of mammographic density on the association of youth body fatness with breast cancer risk in the Nurses’ Health Studies was weak [8].

Increasing evidence points to independent effects of absolute dense and non-dense tissue [15]. While percent breast dense area has been considered a stronger predictor of breast cancer risk than absolute area measures [15], recent evidence suggests that volumetric measures may be more predictive than area measures and, further, that absolute dense volume may be a better indicator of breast cancer risk than percent dense volume [39]. Current BMI has been inversely associated with measurements of absolute dense breast tissue [14, 25] and positively associated with absolute non-dense (fat) tissue [14]. We observed strong inverse associations between childhood and adolescent BMI and young adult dense breast volume but no associations with non-dense volume. These findings suggest the possibility of a direct or indirect influence of body fatness in early life on breast tissue composition, driven by the amount of dense tissue. Further, the observed associations were independent of established reproductive risk factors for breast cancer. In a prior analysis within DISC06, after adjustment for childhood BMI, current BMI was inversely associated with percent breast density via its positive association with absolute non-dense (fat) breast volume, while there was no association of current BMI with absolute dense breast tissue volume [25], providing further evidence that the influence of adiposity on breast tissue composition differs over the life course.

Hypothesized mechanisms for the association between childhood or adolescent body fatness and breast density and cancer risk could include hormonal pathways. For example, levels of some sex steroid hormones may be higher in girls who are heavier [40, 41] and may be associated with earlier differentiation of breast tissue, which could lead to reduced risk of cancer [42, 43]. Body fatness during childhood and adolescence could decrease breast cancer risk via effects on ovarian function. Specifically, obesity suppresses ovarian function, leading to fewer ovulatory menstrual cycles and decreased circulating levels of estrogens and progesterone [44, 45]. Early life body fatness could also influence breast density and cancer risk through altered levels of insulin-like growth factor-1 (IGF-1): increased weight during childhood is directly associated with IGF-1 measured concurrently [46, 47] but early life body size was inversely correlated with IGF-1 levels measured later in adulthood in the Nurses’ Health Study [48], suggesting a possible pathway linking childhood/adolescent body fatness, mammographic density, and breast cancer risk. However, IGF-1 has been inconsistently associated with mammographic density in previous studies [49–55]. Of note, prior studies have demonstrated that childhood adiposity is inversely related to growth velocity [56]. Given that slower growth velocity is associated with lower risk of breast cancer [1, 57], breast density could represent a possible link between these findings and a biologic pathway to breast cancer risk. Finally, other currently unidentified influences of fat on mammary gland development are possible. Consistent with our findings, induced obesity in mice fed a high-fat diet in the pubertal period was associated with stunted mammary ductal growth and reduced mammary epithelial cell proliferation [58]. Interactions of diet and body fatness in early life warrant further investigation [21].

This analysis has some important potential limitations. First, current BMI is a strong negative predictor of breast density, and youth BMI is positively associated with current BMI. Adjustment for current adult BMI substantially attenuated associations between childhood and adolescent BMI and percent breast density. While associations remained statistically significant, we cannot rule out possible residual confounding by current BMI. However, the strong inverse associations between childhood and adolescent BMI and absolute dense breast volume (versus non-dense volume) are not consistent with the idea that early life body fatness is merely a proxy for adult adiposity. Second, strong correlations between BMI measured at successive youth clinic visits make it difficult to disentangle effects at a specific age or developmental period. Although we did not detect evidence of multicollinearity in these models, it remains a potential theoretical concern and may limit our interpretation of age-specific findings and could have contributed to attenuated non-significant findings for absolute non-dense breast area. Third, because the original DISC study population excluded children whose weight-for-height was greater than the 90^th^ percentile or lower than the 5^th^ percentile at baseline [16], our findings may not be generalizable to very lean or very obese children. Finally, only seven women reported a family history of breast cancer at the time of follow-up; because their family members may not have developed breast cancer yet, there is potential misclassification of this variable.

Major strengths of this study include its prospective design and objective and repeated measures of childhood and adolescent weight and height whereas previous studies have been mostly based on retrospective recall and relative body fatness (e.g., somatotype). In addition, we had detailed questionnaire information and the ability to adjust for many potential confounders. Systematic measurement of breast density via MRI afforded us the ability to consider dense versus non-dense breast volume separately, which provides additional information suggesting effects of youth body fatness on breast tissue composition. Finally, this study is among the few studies to evaluate these associations in young premenopausal women ages 25–29; most previous studies focused on postmenopausal women and older premenopausal women.

## Conclusions

In conclusion, these results support the hypothesis that body fatness during childhood and adolescence may play an important role in premenopausal breast density, independent of current adult BMI. Given strong inverse associations with absolute dense tissue volume and lack of association with non-dense volume, our findings further suggest direct or indirect influences of earlier life adiposity on absolute dense breast volume. Further research is warranted to evaluate the possible biological mechanisms underlying these associations and to assess associations with later premenopausal breast density measurements and how changes in breast density over the life course may relate to future breast cancer risk.

## Additional files

Additional file 1: Figure S1a. Distributions of BMI at follow-up, percent breast density, and log-transformed percent breast density. **Figure S1b.** Distributions of absolute dense and non-dense breast volume and log-transformed volume measures. Additional file 2: Table S1. Selected descriptive characteristics over five youth DISC visits. **Table S2.** Pearson correlations of anthropometric characteristics between visits. **Table S3.** Pearson correlation coefficients for measures of breast density and BMI during youth and follow-up.

## References

[1] Ahlgren M, Melbye M, Wohlfahrt J, Sorensen TI (2004). Growth patterns and the risk of breast cancer in women. N Engl J Med.

[2] Bardia A, Vachon CM, Olson JE, Vierkant RA, Wang AH, Hartmann LC (2008). Relative weight at age 12 and risk of postmenopausal breast cancer. Cancer Epidemiol Biomarkers Prev.

[3] Baer HJ, Tworoger SS, Hankinson SE, Willett WC (2010). Body fatness at young ages and risk of breast cancer throughout life. Am J Epidemiol.

[4] McCormack VA, dos Santos SI (2006). Breast density and parenchymal patterns as markers of breast cancer risk: a meta-analysis. Cancer Epidemiol Biomarkers Prev.

[5] Yaffe MJ (2008). Mammographic density. Measurement of mammographic density. Breast Cancer Res.

[6] Samimi G, Colditz GA, Baer HJ, Tamimi RM (2008). Measures of energy balance and mammographic density in the Nurses’ Health Study. Breast Cancer Res Treat.

[7] Sellers TA, Vachon CM, Pankratz VS, Janney CA, Fredericksen Z, Brandt KR (2007). Association of childhood and adolescent anthropometric factors, physical activity, and diet with adult mammographic breast density. Am J Epidemiol.

[8] Harris HR, Tamimi RM, Willett WC, Hankinson SE, Michels KB (2011). Body size across the life course, mammographic density, and risk of breast cancer. Am J Epidemiol.

[9] McCormack VA, dos Santos SI, De Stavola BL, Perry N, Vinnicombe S, Swerdlow AJ (2003). Life-course body size and perimenopausal mammographic parenchymal patterns in the MRC 1946 British birth cohort. Br J Cancer.

[10] Jeffreys M, Warren R, Gunnell D, McCarron P, Smith GD (2004). Life course breast cancer risk factors and adult breast density (United Kingdom). Cancer Causes Control.

[11] Lope V, Perez-Gomez B, Moreno MP, Vidal C, Salas-Trejo D, Ascunce N (2011). Childhood factors associated with mammographic density in adult women. Breast Cancer Res Treat.

[12] Novotny R, Daida Y, Morimoto Y, Shepherd J, Maskarinec G (2011). Puberty, body fat, and breast density in girls of several ethnic groups. Am J Hum Biol.

[13] Lokate M, Peeters PH, Peelen LM, Haars G, Veldhuis WB, van Gils CH (2011). Mammographic density and breast cancer risk: the role of the fat surrounding the fibroglandular tissue. Breast Cancer Res.

[14] Pettersson A, Hankinson SE, Willett WC, Lagiou P, Trichopoulos D, Tamimi RM (2011). Nondense mammographic area and risk of breast cancer. Breast Cancer Res.

[15] Pettersson A, Graff RE, Ursin G, Santos Silva ID, McCormack V, Baglietto L, et al. Mammographic density phenotypes and risk of breast cancer: a meta-analysis. J Natl Cancer Inst. 2014;106: doi:10.1093/jnci/dju078 10.1093/jnci/dju078 PMC4568991 24816206

[16] Dietary intervention study in children (DISC) with elevated low-density-lipoprotein cholesterol. Design and baseline characteristics. DISC Collaborative Research Group. Ann Epidemiol. 1993;3:393–402. 10.1016/1047-2797(93)90067-e 8275216

[17] Efficacy and safety of lowering dietary intake of fat and cholesterol in children with elevated low-density lipoprotein cholesterol. The Dietary Intervention Study in Children (DISC). The Writing Group for the DISC Collaborative Research Group. JAMA. 1995;273:1429–35. 10.1001/jama.1995.03520420045036 7723156

[18] Obarzanek E, Hunsberger SA, Van Horn L, Hartmuller VV, Barton BA, Stevens VJ (1997). Safety of a fat-reduced diet: the dietary intervention study in children (DISC). Pediatrics.

[19] Obarzanek E, Kimm SY, Barton BA, Van Horn LL, Kwiterovich PO, Simons-Morton DG (2001). Long-term safety and efficacy of a cholesterol-lowering diet in children with elevated low-density lipoprotein cholesterol: seven-year results of the Dietary Intervention Study in Children (DISC). Pediatrics.

[20] Kuczmarski RJ, Ogden CL, Grummer-Strawn LM, Flegal KM, Guo SS, Wei R (2000). CDC growth charts: United States. Adv Data.

[21] Dorgan JF, Liu L, Klifa C, Hylton N, Shepherd JA, Stanczyk FZ (2010). Adolescent diet and subsequent serum hormones, breast density, and bone mineral density in young women: results of the Dietary Intervention Study in Children follow-up study. Cancer Epidemiol Biomarkers Prev.

[22] Pettee Gabriel K, Klifa C, Perez A, Kriska AM, High RR, Snetselaar L (2013). Adolescent and young adult exposure to physical activity and breast density. Med Sci Sports Exerc.

[23] ACR practice guideline for the performance of contrast-enhanced magnetic resonance imaging (MRI) of the breast [Amended 2014 (Resolution 39)] http://acr.org/~/media/ACR/Documents/PGTS/guidelines/MRI_Breast.pdf

[24] Klifa C, Carballido-Gamio J, Wilmes L, Laprie A, Lobo C, DeMicco E (2004). Quantitation of breast tissue index from MR data using fuzzy clustering. Conf Proc IEEE Eng Med Biol Soc.

[25] Dorgan JF, Klifa C, Shepherd JA, Egleston BL, Kwiterovich PO, Himes JH (2012). Height, adiposity and body fat distribution and breast density in young women. Breast Cancer Res.

[26] Dorgan JF, Klifa C, Deshmukh S, Egleston BL, Shepherd JA, Kwiterovich PO (2013). Menstrual and reproductive characteristics and breast density in young women. Cancer Causes Control.

[27] Duan N (1983). Smearing estimate: a nonparametric retransformation approach. JASA.

[28] Marquaridt DW (1970). Generalized inverses, ridge regression, biased linear estimation, and nonlinear estimation. Technometrics.

[29] Belsley DA, Kuh E, Welsch RE (1980). Regression diagnostics: identifying influential data and sources of collinearity.

[30] Neter J, Wasserman W, Kutner MH (1989). Applied linear regression models.

[31] Kennedy P (1992). A guide to econometrics.

[32] Hair JFJ, Anderson RE, Tatham RL, Black WC (1995). Multivariate data analysis.

[33] Caire-Juvera G, Arendell LA, Maskarinec G, Thomson CA, Chen Z (2008). Associations between mammographic density and body composition in Hispanic and non-Hispanic white women by menopause status. Menopause.

[34] Rice MS, Bertrand KA, Lajous M, Tamimi RM, Torres-Mejia G, Biessy C (2013). Body size throughout the life course and mammographic density in Mexican women. Breast Cancer Res Treat.

[35] Colditz GA, Frazier AL (1995). Models of breast cancer show that risk is set by events of early life: prevention efforts must shift focus. Cancer Epidemiol Biomarkers Prev.

[36] Russo J, Gusterson BA, Rogers AE, Russo IH, Wellings SR, van Zwieten MJ (1990). Comparative study of human and rat mammary tumorigenesis. Lab Invest.

[37] Russo J, Tay LK, Russo IH (1982). Differentiation of the mammary gland and susceptibility to carcinogenesis. Breast Cancer Res Treat.

[38] Andersen ZJ, Baker JL, Bihrmann K, Vejborg I, Sorensen T, Lynge E (2014). Birth weight, childhood body mass index, and height in relation to mammographic density and breast cancer: a register-based cohort study. Breast Cancer Res.

[39] Shepherd JA, Kerlikowske K, Ma L, Duewer F, Fan B, Wang J (2011). Volume of mammographic density and risk of breast cancer. Cancer Epidemiol Biomarkers Prev.

[40] Baer HJ, Colditz GA, Willett WC, Dorgan JF (2007). Adiposity and sex hormones in girls. Cancer Epidemiol Biomarkers Prev.

[41] Rich-Edwards JW, Goldman MB, Willett WC, Hunter DJ, Stampfer MJ, Colditz GA (1994). Adolescent body mass index and infertility caused by ovulatory disorder. Am J Obstet Gynecol.

[42] Cabanes A, Wang M, Olivo S, DeAssis S, Gustafsson JA, Khan G (2004). Prepubertal estradiol and genistein exposures up-regulate BRCA1 mRNA and reduce mammary tumorigenesis. Carcinogenesis.

[43] Nagasawa H, Yanai R, Shodono M, Nakamura T, Tanabe Y (1974). Effect of neonatally administered estrogen or prolactin on normal and neoplastic mammary growth and serum estradiol-17 beta level in rats. Cancer Res.

[44] Apter D, Vihko R (1990). Endocrine determinants of fertility: serum androgen concentrations during follow-up of adolescents into the third decade of life. J Clin Endocrinol Metab.

[45] Caprio S, Hyman LD, Limb C, McCarthy S, Lange R, Sherwin RS (1995). Central adiposity and its metabolic correlates in obese adolescent girls. Am J Physiol.

[46] Ong K, Kratzsch J, Kiess W, Dunger D (2002). Circulating IGF-I levels in childhood are related to both current body composition and early postnatal growth rate. J Clin Endocrinol Metab.

[47] Velie EM, Zhang Z, Kerver JM, Gardiner JC, Rosen CJ, Dorgan JF (2012). Abstract B78: Adiposity and the IGF-axis in girls during pubertal development. Canc Prev Res.

[48] Poole EM, Tworoger SS, Hankinson SE, Schernhammer ES, Pollak MN, Baer HJ (2011). Body size in early life and adult levels of insulin-like growth factor 1 and insulin-like growth factor binding protein 3. Am J Epidemiol.

[49] Rice MS, Tworoger SS, Rosner BA, Pollak MN, Hankinson SE, Tamimi RM (2012). Insulin-like growth factor-1, insulin-like growth factor-binding protein-3, growth hormone, and mammographic density in the Nurses’ Health Studies. Breast Cancer Res Treat.

[50] Byrne C, Colditz GA, Willett WC, Speizer FE, Pollak M, Hankinson SE (2000). Plasma insulin-like growth factor (IGF) I, IGF-binding protein 3, and mammographic density. Cancer Res.

[51] Aiello EJ, Tworoger SS, Yasui Y, Stanczyk FZ, Potter J, Ulrich CM (2005). Associations among circulating sex hormones, insulin-like growth factor, lipids, and mammographic density in postmenopausal women. Cancer Epidemiol Biomarkers Prev.

[52] Diorio C, Berube S, Byrne C, Masse B, Hebert-Croteau N, Yaffe M (2006). Influence of insulin-like growth factors on the strength of the relation of vitamin D and calcium intakes to mammographic breast density. Cancer Res.

[53] dos Santos SI, Johnson N, De Stavola B, Torres-Mejia G, Fletcher O, Allen DS (2006). The insulin-like growth factor system and mammographic features in premenopausal and postmenopausal women. Cancer Epidemiol Biomarkers Prev.

[54] Bremnes Y, Ursin G, Bjurstam N, Rinaldi S, Kaaks R, Gram IT (2007). Insulin-like growth factor and mammographic density in postmenopausal Norwegian women. Cancer Epidemiol Biomarkers Prev.

[55] Maskarinec G, Takata Y, Chen Z, Gram IT, Nagata C, Pagano I (2007). IGF-I and mammographic density in four geographic locations: a pooled analysis. Int J Cancer.

[56] Berkey CS, Gardner JD, Frazier AL, Colditz GA (2000). Relation of childhood diet and body size to menarche and adolescent growth in girls. Am J Epidemiol.

[57] Berkey CS, Frazier AL, Gardner JD, Colditz GA (1999). Adolescence and breast carcinoma risk. Cancer.

[58] Olson LK, Tan Y, Zhao Y, Aupperlee MD, Haslam SZ (2010). Pubertal exposure to high fat diet causes mouse strain-dependent alterations in mammary gland development and estrogen responsiveness. Int J Obes.

