# Supplementary material for: Body fatness during childhood and adolescence and breast density in young women: a prospective analysis
Source: Breast Cancer Res. 2015 Jul 16;17(1):95. doi: 10.1186/s13058-015-0601-4 (PMC4502611; doi:10.1186/s13058-015-0601-4)
Supplement: Additional file 1: Figure S1a. — Distributions of BMI at follow-up, percent breast density, and log-transformed percent breast density. Figure S1b. Distributions of absolute dense and non-dense breast volume and log-transformed volume measures. [file 13058_2015_601_MOESM1_ESM.docx]

Additional Figure 1a: Distributions of BMI at follow-up, percent breast density, and log-ransformed percent breast density.

Additional Figure 1b: Distributions of absolute dense and non-dense breast volume and log-transformed volume measures.
