# Supplementary material for: Body fatness during childhood and adolescence and breast density in young women: a prospective analysis
Source: Breast Cancer Res. 2015 Jul 16;17(1):95. doi: 10.1186/s13058-015-0601-4 (PMC4502611; doi:10.1186/s13058-015-0601-4)
Supplement: Additional file 2: Table S1. — Selected descriptive characteristics over five youth DISC visits. Table S2. Pearson correlations of anthropometric characteristics between visits. Table S3. Pearson correlation coefficients for measures of breast density and BMI during youth and follow-up. [file 13058_2015_601_MOESM2_ESM.docx]

| **Additional Table 1 Selected descriptive characteristics over 5 youth DISC visits** | | | | |  |
| --- | --- | --- | --- | --- | --- |
|  | Baseline | Year 1 | Year 3 | Year 5 | Last |
| N | 182 | 173 | 171 | 154 | 160 |
| Age (years) | 9.1 (0.6) | 10.2 (0.6) | 12.2 (0.6) | 14.2 (0.6) | 16.6 (0.9) |
| Tanner stage | 1 (0) | 1.6 (0.66) | 2.9 (1.1) | 4.1 (0.85) | 4.9 (0.25) |
| Height (cm) | 133.2 (6.2) | 140.3 (6.6) | 153.2 (7.2) | 161.9 (6.6) | 164.3 (6.2) |
| BMI Z-score | 0.23 (0.90) | 0.27 (0.92) | 0.34 (0.96) | 0.40 (0.90) | 0.34 (0.91) |
| Moderate- to vigorous-intensity physical activity (MET-hrs/week) | 44.3 (38.7) | 58.9 (58.7) | 59.8 (46.7) | 77.4 (59.8) | 91.5 (72.7) |
| Total energy intake (kcal) | 1654 (391) | 1554 (360) | 1618 (451) | 1657 (449) | 1654 (544) |
| Values are Means (SD) |  |  |  |  |  |

| **Additional Table 2 Pearson correlations of anthropometric characteristics between visits** | | | | | | | | |
| --- | --- | --- | --- | --- | --- | --- | --- | --- |
|  | Height at adult follow-up (cm) | BMI at adult follow-up (kg/m2) | BMI Z-score at 8-10 years old (baseline) | BMI Z-score at Year 1 visit | BMI Z-score at Year 3 visit | BMI Z-score at Year 5 visit | BMI Z-score at Last visit |  |
| Height (cm) at adult follow-up | 1.00 | -0.13 | 0.08 | 0.10 | -0.01 | -0.03 | 0.01 |  |
| BMI (kg/m2) at adult follow-up |  | 1.00 | **0.52** | **0.54** | **0.61** | **0.70** | **0.69** |  |
| BMI Z-score at 8-10 years old (baseline) |  |  | 1.00 | **0.94** | **0.87** | **0.81** | **0.74** |  |
| BMI Z-score at Year 1 visit |  |  |  | 1.00 | **0.91** | **0.84** | **0.76** |  |
| BMI Z-score at Year 3 visit |  |  |  |  | 1.00 | **0.90** | **0.76** |  |
| BMI Z-score at Year 5 visit |  |  |  |  |  | 1.00 | **0.88** |  |
| BMI Z-score at Last visit |  |  |  |  |  |  | 1.00 |  |
| **bold:** p-value <0.001 |  |  |  |  |  |  |  |  |

| **Additional Table 3 Pearson correlation coefficients for measures of breast density and BMI during youth and follow-up** | | | | | | |
| --- | --- | --- | --- | --- | --- | --- |
|  | BMI Z-score at 8-10 years old (baseline) | BMI Z-score at Year 1 visit | BMI Z-score at Year 3 visit | BMI Z-score at Year 5 visit | BMI Z-score at Last visit | BMI at adult follow-up (kg/m2) |
| Average percent density | -0.49 | -0.49 | -0.60 | -0.66 | -0.60 | -0.66 |
| Absolute dense volume | -0.35 | -0.37 | -0.42 | -0.45 | -0.36 | -0.25 |
| Absolute non-dense volume | 0.40 | 0.39 | 0.51 | 0.61 | 0.60 | 0.81 |
